# Postnatal growth outcomes and influence of maternal gestational weight gain: a prospective cohort study in rural Vietnam

**DOI:** 10.1186/1471-2393-14-339

**Published:** 2014-09-30

**Authors:** Sarah Hanieh, Tran T Ha, Julie A Simpson, Tran T Thuy, Nguyen C Khuong, Dang D Thoang, Thach D Tran, Tran Tuan, Jane Fisher, Beverley-Ann Biggs

**Affiliations:** Department of Medicine, University of Melbourne, L 4 Clinical Science Building, Royal Melbourne Hospital, Parkville, Victoria 3050 Australia; Research and Training Centre for Community Development (RTCCD), Hanoi, Vietnam; Centre for Molecular, Environmental, Genetic and Analytic Epidemiology, Melbourne School of Population and Global Health, University of Melbourne, Parkville, Victoria Australia; Provincial Centre of Preventive Medicine, Hanam Province, Vietnam; The Jean Hailes Research Unit, School of Public Health and Preventive Medicine, Monash University, Clayton, Victoria Australia; The Victorian Infectious Diseases Service, Royal Melbourne Hospital, Parkville, Victoria Australia

**Keywords:** Gestational weight gain, Postnatal growth, Body mass index

## Abstract

**Background:**

Suboptimal weight gain during pregnancy may result in adverse outcomes for both the mother and child, including increased risk of pre-eclampsia and gestational diabetes, delivery of low birth weight and small-for-gestational age (SGA) infants, and preterm delivery. The objectives of this study were to identify maternal predictors of rate of weight gain in pregnancy, and to evaluate the association of gestational weight gain with infant postnatal growth outcomes.

**Methods:**

We conducted a prospective cohort study of infants born to women who had previously participated in a double-blind cluster randomized controlled trial of antenatal micronutrient supplementation, in Ha Nam province, Vietnam. Pregnant women (n = 1258) were seen at enrolment and 32 weeks gestation, and infants (n = 965) were followed until 6 months of age. Primary outcome was infant anthropometric indicators at 6 months of age (weight for age, length for age, weight for height z scores), and infant weight gain velocity during the first 6 months of life.

**Results:**

Low body mass index (<18.5 kg/m^2^) was present in 26% of women, and rate of gestational weight gain was 0.4 kg per week [SD 0.12]. Rate of weight gain during pregnancy was significantly associated with infant weight-for-age (MD 1.13, 95% CI 0.58 to 1.68), length-for-age (MD 1.11, 95% CI 0.66 to 1.55), weight-for-length z scores (MD 0.63, 95% CI 0.07 to 1.19), and infant weight gain velocity during the first 6 months of life (MD 93.6 g per month, 95% CI 8.2 to 179.0).

**Conclusions:**

Rate of gestational weight gain is predictive of postnatal growth at six months of age in this setting. Public health programs should be targeted towards improving body mass index and weight gain in pregnant women in rural Vietnam.

## Background

Women who enter pregnancy with a sub-optimal body mass index (BMI) and gain either too little or too much weight have an increased risk of delivery of low birth weight and small-for-gestational age (SGA) infants, intra-uterine growth restriction, neonatal mortality, preterm delivery, still birth and congenital defects [1–6]. Limited studies have examined the effect of gestational weight gain extending past the neonatal period into infancy or childhood, and those that are available have been conducted in high-income countries [7–10]. Mounting evidence suggests that growth patterns in early life reflect body composition in adulthood [11], and are associated with increased risk of metabolic syndrome and the development of chronic diseases such as hypertension, coronary heart disease, and high cholesterol [12–14].

Currently the recommended weekly weight gain during the second and third trimesters of pregnancy is 0.8 to 1 kgs/week for women with a normal pre-pregnancy BMI (18.5–24.9 kg/m^2^), 1 to 1.3 kgs/week for women who are underweight (pre-pregnancy BMI < 18.5 kg/m^2^), and 0.5 to 0.7 kgs/week for women who are overweight (pre-pregnancy BMI ≥25 kg/m^2)^ [15]. However these recommendations are based on data collected from developed countries, where the main focus is on the adverse consequences of excess weight gain during pregnancy.

In Vietnam, underweight prevalence remains significantly higher than in other South East Asian countries, with around 20% of women reported to have low BMI (<18.5 kg/m^2^) [16]. Determining predictors of gestational weight gain, and its influence on early infant postnatal growth, would provide an opportunity to better understand the nutritional programming of body composition in resource poor settings. This may enable appropriate targeting of preventive measures in early or pre-pregnancy, to improve child growth and development and reduce disease risk in later life.

We therefore conducted a prospective cohort study in a rural province of Vietnam that aimed to identify maternal predictors of rate of weight gain in pregnancy, and to determine the influence of rate of weight gain during pregnancy on infant growth outcomes at birth and 6 months of age.

## Methods

### Study design

This was an observational cohort study of pregnant women who had previously participated in a double-blind cluster randomized controlled trial of antenatal micronutrient supplementation in rural Vietnam. All women and infants enrolled in the original cluster randomised trial (ACTNR 12610000944033) were eligible for enrolment in the study. Our primary outcomes of interest were infant anthropometric measures at 6 months of age: weight for age z scores (WAZ), length for age z scores (LAZ), weight for length z scores (WLZ), and infant weight gain velocity during the first 6 months of life. Secondary outcomes were birth weight, length, and head circumference.

### Study setting and participants

The study was undertaken in Ha Nam province in northern Vietnam. Ha Nam has a population of approximately 820,100 people, with most residents still working in subsistence agriculture. Diet consists mainly of rice, with some vegetables and meat. In 2010, the average annual per capita income was $ USD 800 [17].

Eligible participants were all women and infants previously enrolled in a cluster randomised trial which took place between 28th September 2010 and 8th Jan 2012. For the original cluster randomised trial, women were randomised to receive either (1) one tablet of iron-folic acid (IFA) taken daily (60 mg elemental iron /0.4 mg folic acid per tablet, 7 tablets per week); (2) one capsule of IFA taken twice a week (60 mg elemental iron /1.5 mg folic acid per capsule; 2 capsules per week); or (3) one capsule of multiple micronutrients (MMN) taken twice a week (60 mg elemental iron/1.5 mg folic acid per capsule/ plus zinc 20 mg, iodine 300 ug, copper 4 mg, selenium 130 ug, niacin 36 mg, Vitamins A 1.6 mg, B1 2.8 mg, B2 2.8 mg, B12 5.2 ug, C 140 mg, D 400 IU, E 20 mg;2 capsules per week.) Supplements were taken from enrolment until 3 months post-partum and primary outcome was infant birthweight. Each woman was visited every 6 weeks at home, to distribute the intervention with written instructions for the next 6 weeks, and to collect information on adherence, side effects, and pregnancy complications. The median level of adherence was 91% in the daily IFA group, 96% in the twice weekly IFA group, and 85% in the twice weekly MMN group. Adherence was verified with direct questioning, and by checking the number of tablets remaining. This trial has been previously published [18].

### Data collection and outcomes

Rate of weight gain during pregnancy was derived from weight measurements taken at enrolment (range 2–16 weeks; mean gestational age 12.2 weeks) and at 32 weeks gestation (range 31–39 weeks; mean gestational age 33.4 weeks), divided by the number of weeks between the two observations. Infants were seen at birth and 6 months of age.

### Maternal characteristics

Maternal sociodemographic factors were assessed using a questionnaire administered by trained research staff at enrolment. With early pregnancy (first trimester) dietary history, women were asked if they had changed their dietary habits (eating more vegetables, eating more nutritiously or eating larger quantities of food) on finding out they were pregnant. Meat intake (number of times per week) and use of prenatal nutritional or traditional supplementation were also recorded.

### Maternal anthropometric measurements

Maternal height was measured with a portable stadiometer (Seca 214, Hamburg, Germany), and maternal weight with a mother-infant scale (Seca 872, Hamburg, Germany). BMI was calculated as weight in kilograms divided by height in metres squared. Mid upper arm circumference (MUAC) was measured using a flexible measuring tape at enrolment. Low mid upper arm circumference was defined as MUAC < 23.5 cm and short maternal stature was defined as height < 145 cm.

### Infant anthropometric measurements

Infant crown-heel length was measured using a portable Shorr Board (Shorr productions, Olney Md. USA), and weight was measured using electronic SECA 876 scales (SECA Ltd. Germany), with precision to the nearest 100 g. Infant weight for age, weight for length and length for age z scores were calculated using WHO Anthro (version 3.2.2, January 2011) [19]. Stunting was defined as length-for-age z scores less than two standard deviations below WHO Child Growth Standards, wasting as weight for length z scores less than two standard deviations below WHO Child Growth Standards, underweight as weight for age z scores less than two standard deviations below WHO Child Growth Standards, and low mid-upper arm circumference as mid upper arm circumference z scores less than two standard deviations below WHO Child Growth Standards [20]. Infant weight gain velocity was derived from weight measurements taken at birth and 6 months of age, divided by the number of months between the two observations.

### Gestational age

Gestational age at birth was calculated from estimated gestational age recorded by trans-abdominal ultrasound performed at the district hospital if available (1041 (82.8%) mothers), and if not, according to the date of last menstruation (136(10.8%) mothers).

### Ethics considerations

The study was approved by the Melbourne Health Human Research Ethics Committee, and the Hanam Provincial Human Research Ethics Committee. The original cluster randomised trial was registered in the Australia New Zealand Clinical Trials Registry: 12610000944033. Written informed consent was collected from all participants prior to enrolment.

### Statistical analysis

Data were analysed using Stata, Version 12 (StataCorp, College Station, TX, USA). Categorical data are presented as percentages with frequency, and continuous data are presented as mean and standard deviation (SD). Univariable linear regression was performed to examine the association between 1) maternal factors (demographic, anthropometric, and antenatal factors) associated with rate of weight gain during pregnancy, and 2) the association between rate of weight gain during pregnancy and infant anthropometeric outcomes (birthweight, length, head circumference, WAZ, WHZ, HAZ scores and infant weight gain velocity at 6 months of age). Statistically significant predictors (defined as p < 0.05 in univariable analysis) were then selected for inclusion in a multivariable model along with potential confounding factors (. Unadjusted and adjusted estimates of mean differences or odds ratios, and their 95% confidence intervals (CI) are presented.

## Results

**Figure 1:**
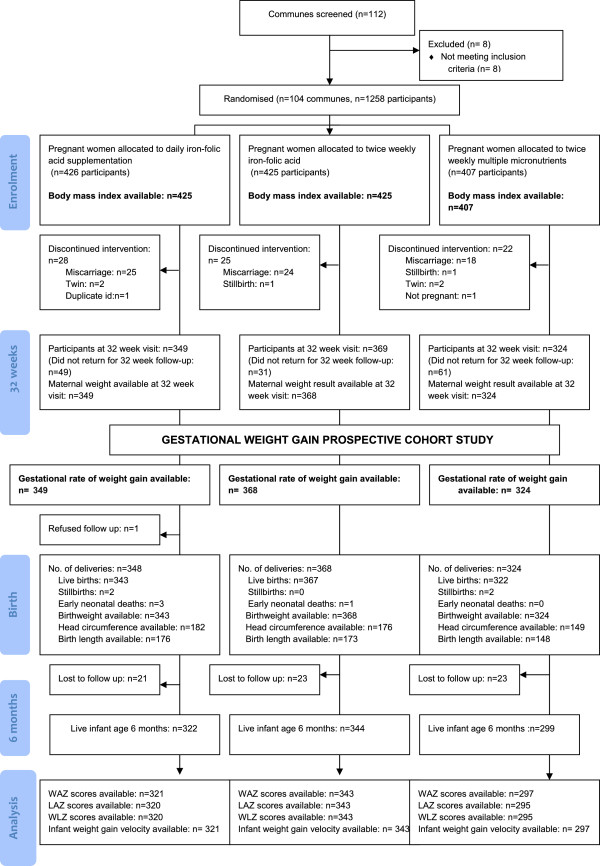
**Flow diagram.**

The study flow diagram is presented in Figure 1. A total of 1258 pregnant women were enrolled into the original cluster randomised trial, 1042 women were seen at 32 weeks, and gestational rate of weight gain was available on 1041 women (82.8%). 965 infants were seen at the 6 month visit. The three arms of the trial were equally represented among infants followed until 6 months of age. Twenty-six percent (329/1257) of women were underweight at baseline (BMI < 18.5 kg/m^2^), and 1.4% (18/1257) were classified as overweight (BMI > 30 kg/m^2^). Average weekly weight gain was 0.4 kg/week [SD 0.13].

Baseline maternal characteristics according to baseline maternal BMI status are presented in Table 1. Mid upper arm circumference, meat intake during pregnancy and use of traditional supplements in early pregnancy were associated with maternal BMI at baseline.

**Table 1:** **Baseline characteristics of pregnant women in rural Vietnam by grouping of BMI measurement at enrolment (<18.5, ≥18.5 kg/m** ^**2**^ **)**

Maternal characteristic	Total ^*^(n = 1257)	[SD] or (%)	Underweight ^*^(BMI < 18.5 kg/m ^2^) (n = 329)	[SD] or (%)	Not underweight ^*^(BMI > =18.5 kg/m ^2^) (n = 928)	[SD] or (%)	P value†
**Demographic factors**							
Age (years)	26.8	[5.0]	26.4	[4.7]	26.9	[5.1]	0.07
Educational level							
Primary school	160	(15.3)	47	(14.3)	151	(16.3)	0.64
Secondary school	705	(67.2)	216	(65.6)	603	(65.1)	
University/college	184	(17.5)	66	(20.1)	173	(18.7)	
Occupation							0.83
Farmer/housewife	653	(52.0)	168	(51.1)	484	(52.2)	
Factory worker/trader	418	(33.3)	109	(33.1)	309	(33.3)	
Government official/clerk	186	(14.8)	52	(15.8)	134	(14.5)	
**Anthropometric factors**							
Height (cm)	153.6	[4.8]	153.9	[0.26]	153.5	[0.16]	0.23
Mid upper arm circumference enrolment (cm)	23.8	[2.05]	21.9	[1.28]	24.5	[1.82]	<0.001
**Antenatal factors**							
Gravidity							0.21
Primigravida	386	(30.7)	110	(33.4)	276	(29.7)	
Multigravida	871	(69.3)	219	(66.6)	652	(70.3)	
Change of diet when pregnant	941	(74.9)	258	(78.4)	683	(73.7)	0.09
Meat intake during pregnancy at enrolment (no. of times per week)	3.84	[2.28]	3.60	[2.33]	3.92	[2.26]	0.03
Use of nutritional supplements at baseline	699	(55.6)	190	(57.8)	508	(54.8)	0.36
Use of traditional supplements at baseline	57	(4.5)	22	(6.7)	35	(3.8)	0.03
Depression EPDS	235	(18.7)	71	(21.6)	164	(17.7)	0.12

^*^Values are mean or number.

†P value determined from chi squared or students t-test.

### Factors associated with rate of weight gain during pregnancy

Maternal factors associated with rate of weight gain during pregnancy were demographic: education (university/college educated vs primary school- MD 0.03 kg/week, 95% CI 0.01 to 0.06), occupation (government worker vs farmer- MD 0.06 kg/week, 95% CI 0.03 to 0.09); taking over the counter nutritional supplements in early pregnancy (0.02 kg/week, 95% CI 0.01 to 0.03), and low mid upper arm circumference at baseline (MD 0.02 kg/week, 95% CI 0.01 to 0.03). The most commonly used over the counter supplements were iron supplements (71.4%). Factors inversely associated with rate of weight gain during pregnancy included baseline BMI (-0.01 kg per kg/week, 95% CI -0.01 to -0.004), and short maternal stature (MD -0.05 kg/week, 95% CI -0.09 to -0.02) (Table 2).

**Table 2:** **Predictors of rate of weight gain (kg/week) during pregnancy (univariable and multivariable regression)**

Maternal characteristic	Univariable regression			Multivariable regression*		
	Coefficient	(95% CI)	P value	Coefficient	(95% CI)	P value
**Demographic factors**						
Maternal age (per 10 years)	-0.01	(-0.03 to 0.01)	0.09			
Educational level						
Primary school	Reference		-	Reference		-
Secondary school	0.01	(-0.01 to 0.03)	0.50	-0.01	(-0.02 to 0.02)	0.93
University/College	0.08	(0.06 to 0.11)	<0.001	0.03	(0.004 to 0.06)	0.03
Occupation^2^						
Farmer/housewife	Reference		-	Reference		-
Factory worker/trader	0.01	(-0.01 to 0.03)	0.24	0.01	(-0.01 to 0.02)	0.61
Government official/clerk	0.09	(0.07 to 0.12)	<0.001	0.06	(0.03 to 0.09)	<0.001
**Anthropometric factors**						
Low mid upper arm circumference (<23.5 cm)^3^	0.02	(0.01 to 0.04)	0.01	0.02	(0.01 to 0.03)	0.04
Maternal body mass index at enrolment (kg/m^2^)	-0.01	(-0.02 to -0.04)	<0.001	-0.01	(-0.01 to -0.004)	0.001
Short maternal stature (height < 145 cm)	-0.05	(-0.10 to -0.01)	0.02	-0.05	(-0.09 to -0.02)	0.01
**Antenatal factors**						
Gravidity						
Primigravida	Reference		-	Reference		-
Multigravida	-0.03	(-0.05 to -0.01)	<0.001	-0.01	(-0.03 to 0.01)	0.21
Depression at enrolment (EPDS)	-0.01	(-0.03 to 0.01)	0.54			
**Nutritional factors**						
Taking nutritional supplementation	0.04	(0.03 to 0.06)	<0.001	0.02	(0.01 to 0.03)	0.03
Taking traditional supplements	-0.01	(-0.05 to 0.03)	0.77			
Meat intake during pregnancy at enrolment (no. times per week)	0.01	(0.001 to 0.006)	0.09			
Change of diet in early pregnancy	0.03	(0.01 to 0.04)	0.01	0.01 (-0.01 to 0.03)		0.05
Type of supplement taken during pregnancy						
Daily IFA supplements	Reference		-	Reference		-
Twice weekly IFA supplements	0.01	(-0.02 to 0.02)	0.83	0.01	(-0.02 to 0.02)	0.93
MMN supplements	-0.01	(-0.03 to 0.01)	0.31	-0.01	(-0.02 to 0.01)	0.59

^*^Model adjusted for supplement group, gestational age at enrolment and cluster randomisation.

### Association of rate of weight gain during pregnancy with infant birth outcomes

Rate of gestational weight gain was significantly associated with infant birthweight (MD 532.3 gms, 95% CI 339.8 to 724.9) and birth length (MD 2.41 cm, 95% CI 0.49 to 4.33), but not with head circumference (MD 0.35, 95% CI -1.21 to 1.91) (Table 3).

**Table 3:** **Associations between rate of weight gain (kg/week) during pregnancy and birth outcomes (univariable and multivariable regression)**

Birth outcomes			Unadjusted			Adjusted ^*^		
	Mean or number	[SD] or (%)	Coefficient	(95% CI)	P value	Coefficient	(95% CI)	P value
Birthweight (grams)	3169.4	[391.4]	479.0	(290.3 to 667.6)	<0.001	532.3	(339.8 to 724.9)	<0.001
Head circumference (cm)	32.7	[2.11]	0.38	(-1.17 to 1.94)	0.63	0.35	(-1.21 to 1.91)	0.66
Length (cm)	49.1	[2.59]	2.29	(0.38 to 4.20)	0.02	2.41	(0.49 to 4.33)	0.02

^*^Adjusted for maternal education, maternal body mass index, supplement arm, and cluster randomisation.

### Association of rate of weight gain during pregnancy with infant anthropometric outcomes at 6 months of age

Stunting prevalence was 6.4% at 6 months of age, and prevalence of wasting was less than 2%. A significant association between rate of gestational weight gain and infant weight-for-age (1.35, 95% CI 0.78 to 1.92), length-for-age (1.18, 95% CI 0.72 to 1.64), and weight-for-length z scores (MD 0.84, 95% CI 0.25 to 1.43) was demonstrated (Table 4).

**Table 4:** **Associations between rate of weight gain (kg/week) during pregnancy and infant anthropometric outcomes (univariable and multivariable regression)**

Anthropometric outcome at 6 months of age			Unadjusted			Adjusted ^*^		
	Mean	[SD]	Coefficient	(95% CI)	P value	Coefficient	(95% CI)	P value
Weight for age z score	-0.10	[0.10]	1.03	(0.53 to 1.53)	<0.001	1.35	(0.78 to 1.92)	<0.001
Weight for length z score	0.40	[1.01]	0.51	(-0.01 to 1.03)	0.05	0.84	(0.25 to 1.43)	0.01
Length for age z score	-0.57	[0.92]	1.09	(0.63 to 1.56)	<0.001	1.18	(0.72 to 1.64)	<0.001
Weight gain velocity (gm/month)	717.6	[141.6]	79.0	(7.32 to 150.8)	0.03	113.5	(26.3 to 200.6)	0.01

^*^Adjusted for maternal education, maternal body mass index, supplement arm, gestational age at enrolment, wealth index and cluster randomisation.

### Association of rate of weight gain during pregnancy with infant weight gain velocity

Mean infant weight gain velocity was 717.6 grams /month, SD [141.6]. For every 1 kg/week increase in weight gain during pregnancy, infant weight gain velocity increased by 93.6 grams per month (95% CI 8.2 to 179.0) during the first 6 months of life.

## Discussion

Our findings indicate that rate of gestational weight gain was significantly associated with infant birth (weight and length) and growth outcomes at 6 months of age (weight for age, length for age, weight for length z scores weight gain velocity). We also documented a high prevalence of pregnant women residing in rural Ha Nam Province, Vietnam with a low BMI in early pregnancy. Maternal tertiary education and office-based occupation, as well as use of nutritional supplementation in early pregnancy predicted increased rate of weight gain during pregnancy. To our knowledge, this is the first study in a lower-income country to examine the influence of rate of gestational weight gain on postnatal growth outcomes.

Growth patterns in early infancy have been shown to make a significant contribution to body composition in later life, which may influence the future health and disease development in the child [11, 21]. Previous studies have demonstrated that undernutrition during the *in utero* period can permanently change the physiology and composition of the body either through direct effects on the cell, or as a result of altered concentrations of growth factors, hormones, distribution of cell types, metabolic activity and organ structure. These changes can then in turn increase the risk of disease such as coronary heart disease, stroke, diabetes and hypertension in adult life [12, 22–26].

The majority of previous studies in the literature have focussed on the association between weight gain during pregnancy and outcomes at birth [1–4, 27–29]. Using data from seven population based cohort studies, Black et al. have shown that low maternal BMI during the pregnancy period significantly increases the risk of having a small for gestational age baby [30]. In a large cohort study in Vietnam, Ota et al. estimated the risk of giving birth to an infant too small or too large for gestational age as a function of maternal gestational weight gain and BMI, and demonstrated that a low maternal BMI and a weight gain of < 10 kg during pregnancy significantly increased the risk of delivering a small for gestational age baby [3]. Liu et al. found that women who were overweight/obese and had high weight gain, as well as those who were underweight and had a low weight gain, had a significantly higher risk of adverse pregnancy outcomes including large or small for gestational age, low birth weight and pre-eclampsia in a large cohort of singleton term pregnancies (n = 292, 568) [31]. Our findings extend those of Heerman et al. who demonstrated that the combined effect of pre-pregnancy BMI and maternal gestational weight gain was significantly associated with infant growth trajectory throughout the first year of life [32]. We also demonstrated an inverse association between baseline maternal body mass index and rate of gestational weight gain, and this has also been shown by Rode et al. who found that gestational weight gain decreased with increasing body mass index [33].

Undernutrition at different times during the gestational period has been shown to result in varying effects on growth [12, 22–25]. Barker et al. demonstrated that undernutrition during late gestation leads to disproportionate growth, whilst undernutrition during early gestation leads to proportionate loss of body size [22]. Disproportionate growth has been shown to result in an increased risk of coronary artery disease and other non-communicable diseases [23]. Our findings strengthen the need for an urgent public health focus on improving the nutrition of pregnant women in Vietnam, and this is of particular importance in lower and middle income countries where overweight is an emerging problem and morbidity and mortality from non-communicable diseases is increasing [34].

In our study, we demonstrated a direct link between rate of weight gain during pregnancy and early infant weight gain velocity. Emerging evidence has shown that children who are undernourished or have poor weight gain during the first 2 years of life, are at significantly higher risk of chronic diseases in adulthood [14, 35, 36]. For example Hale et al. have previously proposed a thrifty ‘phenotype hypothesis’ where poor nutrition in fetal and early infant life predisposes to the later development of Type 2 diabetes through defects in the structure and function of Beta cells of the islets of Langerhans [37, 38]. Further follow up of our cohort of infants beyond the early infancy period will therefore be important, to determine the long-term adverse consequences related to inadequate gestational weight gain in this setting.

We found that nearly 30% of women had low BMI in early pregnancy, which is an indicator of chronic energy deficiency and has been shown to be related to body fat mass and fat-free mass [39]. Although the prevalence of low maternal BMI has declined globally over the past twenty years, maternal under-nutrition is still of major concern in Asia and Africa [30]. Vietnam has achieved significant and rapid economic growth in the last decade, resulting in a reduction in the prevalence of the total population living under the poverty line, and a transformation in nutritional profile of the country [17]. Over the last two decades, a significant increase in the consumption of meat, total fat and eggs among the population has been documented, and underweight prevalence (BMI <18.5 kg/m^2^) in women aged 20–49 years has reduced from 33.1% in 1990 to 20.5% in 2005 [16]. However, the rapid economic growth within the country has served to widen the socio-economic gap, with many of the rural population still facing significant inequities in economic, development and health burdens.

The strengths of our study are that infant anthropometric outcomes were followed past the neonatal period to six month of age, and that we measured BMI and weight gain directly, rather than rely on participant self-reporting as in many previous studies. In addition, our study was conducted in a rapidly developing rural area, representative of many areas of Vietnam, and thus our findings may be generalizable to other parts of the country.

Limitations of our study are that weight during pregnancy was only measured at enrolment and during late pregnancy (range 31–39 weeks; mean gestational age 33.4 weeks), and therefore we were unable to estimate total weight gain during pregnancy; and that maternal early pregnancy dietary habits were not accurately quantified. However despite this, the large sample size, and rigorous trial design of the original cluster randomised controlled trial, have allowed us to measure a comprehensive set of factors during the pregnancy period that predict rate of gestational weight gain, and to determine the critical influence of rate of weight gain during pregnancy on infant anthropometric outcomes.

## Conclusions

Our findings highlight the important issue of low BMI in early pregnancy and the influence of gestational weight gain on infant outcomes in rural Vietnam. Rate of weight gain during pregnancy was found to be significantly associated with postnatal growth at six months of age, which may have adverse consequences for the infant that extend into childhood and beyond. Public health programs should be targeted towards improving nutritional status and weight gain in pregnant women in rural Vietnam.
